# 3,3′-Oxybi[isobenzofuran-1(3*H*)-one]

**DOI:** 10.1107/S1600536809038914

**Published:** 2009-09-30

**Authors:** Wenkuan Li, Handong Yin, Liyuan Wen, Kang Li, Weidong Fan

**Affiliations:** a College of Chemistry and Chemical Engineering, Liaocheng University, Shandong 252059, People’s Republic of China

## Abstract

The title compound, C_16_H_10_O_5_, consists of two isobenzofuran-1(3*H*)-one moieties which are linked by a bridging O atom. The two halves of the mol­ecule display approximate non-crystallographic mirror symmetry. The dihedral angle between the two isobenzofuran-1(3*H*)-one ring systems is 53.18 (6) Å. Two chiral carbon centres are observed in the compound, but their absolute configurations could not be determined. In the crystal structure, inter­molecular C—H⋯O hydrogen bonds link mol­ecules into zigzag chains along *c*. Additional C—H⋯O inter­actions connect adjacent chains.

## Related literature

For general background to isobenzofuran-1(3*H*)-ones, see: Landge *et al.* (2008 ▶); Mukhopadhyay & Kundu (2001 ▶); Paradkar *et al.* (1998 ▶).
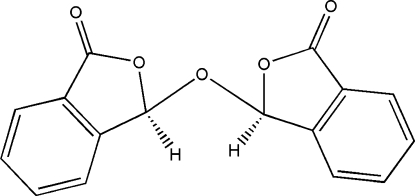


## Experimental

### 

#### Crystal data

C_16_H_10_O_5_
*M* *_r_* = 282.24
Monoclinic, 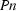

*a* = 4.4449 (6) Å
*b* = 6.4937 (8) Å
*c* = 22.222 (2) Å
β = 91.334 (1)°
*V* = 641.25 (13) Å^3^
*Z* = 2
Mo *K*α radiation
μ = 0.11 mm^−1^
*T* = 298 K
0.44 × 0.29 × 0.11 mm

#### Data collection

Siemens SMART CCD area-detector diffractometer
Absorption correction: multi-scan (*SADABS*; Sheldrick, 1996 ▶) *T*_min_ = 0.953, *T*_max_ = 0.988
3081 measured reflections
1139 independent reflections
972 reflections with *I* > 2σ(*I*)
*R*_int_ = 0.025

#### Refinement

*R*[*F* ^2^ > 2σ(*F* ^2^)] = 0.031
*wR*(*F* ^2^) = 0.078
*S* = 1.01
1139 reflections
190 parameters
2 restraints
H-atom parameters constrained
Δρ_max_ = 0.13 e Å^−3^
Δρ_min_ = −0.13 e Å^−3^

### 

Data collection: *SMART* (Siemens, 1996 ▶); cell refinement: *SAINT* (Siemens, 1996 ▶); data reduction: *SAINT*; program(s) used to solve structure: *SHELXS97* (Sheldrick, 2008 ▶); program(s) used to refine structure: *SHELXL97* (Sheldrick, 2008 ▶); molecular graphics: *SHELXTL* (Sheldrick, 2008 ▶); software used to prepare material for publication: *SHELXTL*.

## Supplementary Material

Crystal structure: contains datablocks I, global. DOI: 10.1107/S1600536809038914/sj2658sup1.cif

Structure factors: contains datablocks I. DOI: 10.1107/S1600536809038914/sj2658Isup2.hkl

Additional supplementary materials:  crystallographic information; 3D view; checkCIF report

## Figures and Tables

**Table 1:** Hydrogen-bond geometry (Å, °)

*D*—H⋯*A*	*D*—H	H⋯*A*	*D*⋯*A*	*D*—H⋯*A*
C6—H6⋯O5^i^	0.93	2.68	3.407 (4)	136
C1—H1⋯O3^ii^	0.98	2.61	3.337 (4)	131

Symmetry codes: (i) 
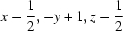
; (ii) 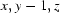.

**Fig. 1.**
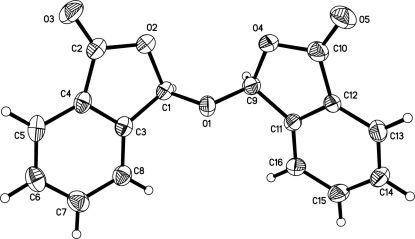
The asymmetric unit of the title compound, showing 50% probability displacement ellipsoids.

**Fig. 2.**
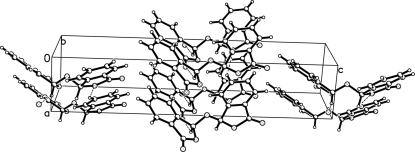
Packing diagram of the title compound viewed approximately along the b axis. H atoms are omitted for clarity.

## supplementary crystallographic information

### Comment

Isobenzofuran-1(3*H*)-ones represent an important class of natural products that possess significant biological properties (Landge *et al.*, 2008). A  number of isobenzofuran derivatives have been reported in the literature (Paradkar *et al.*, 1998; Mukhopadhyay & Kundu, 2001). Herein, the crystal structure of the title compound is presented.

The title compound (Fig. 1) is composed of two isobenzofuran-1(3*H*) -one moieties which are linked together by a bridging O1 atom. The two moieties display approximate mirror symmetry. The two isobenzofuran-1(3*H*)-one ring systems are not coplanar and are twisted from each other by a dihedral angle of 53.18 (6)Å. The title compound is a chiral compound with two chiral centers at C_1_ and C_9_ but the absolute configuration could not be determined. In the crystal structure, C6—H6···O5 hydrogen bonds link molecules into zig-zag chains along *c*.  Additional C1—H1···O3 interactions connect adjacent chains (Fig. 2).

### Experimental

The product was obtained by a simple synthetic method: A mixture of triphenylantimony (3.00 mmol) and phthalic acid (3.00 mmol) in a stoppered flask was heated to 383 K. The mixture was shaken for 2.5 h. The product was crytallized from a solution of dichloromethane/petroleum ether (1:1), and to afford the title compound unexpectedly.

### Refinement

In the absence of significant anomalous scattering effects, 1139 Friedel pairs were merged. H atoms were positioned geometrically, and constrained to ride on their parent atoms, with C—H = 0.93 and 0.98 Å for aromatic and methine H atoms, respectively. and with *U*_iso_(H) = 1.2*U*_eq_(C).

### Crystal data

**Table:**

C_16_H_10_O_5_	*F*(000) = 292
*M**_r_* = 282.24	*D*_x_ = 1.462 Mg m^−^^3^
Monoclinic, *P**n*	Mo *K*α radiation, λ = 0.71073 Å
Hall symbol: -P 2yac	Cell parameters from 1280 reflections
*a* = 4.4449 (6) Å	θ = 2.8–23.0°
*b* = 6.4937 (8) Å	µ = 0.11 mm^−^^1^
*c* = 22.222 (2) Å	*T* = 298 K
β = 91.334 (1)°	Block, colorless
*V* = 641.25 (13) Å^3^	0.44 × 0.29 × 0.11 mm
*Z* = 2	

### Data collection

**Table:**

Siemens SMART CCD area-detector diffractometer	1139 independent reflections
Radiation source: fine-focus sealed tube	972 reflections with *I* > 2σ(*I*)
graphite	*R*_int_ = 0.025
φ and ω scans	θ_max_ = 25.0°, θ_min_ = 1.8°
Absorption correction: multi-scan (*SADABS*; Sheldrick, 1996)	*h* = −3→5
*T*_min_ = 0.953, *T*_max_ = 0.988	*k* = −7→7
3081 measured reflections	*l* = −26→26

### Refinement

**Table:**

Refinement on *F*^2^	Primary atom site location: structure-invariant direct methods
Least-squares matrix: full	Secondary atom site location: difference Fourier map
*R*[*F*^2^ > 2σ(*F*^2^)] = 0.031	Hydrogen site location: inferred from neighbouring sites
*wR*(*F*^2^) = 0.078	H-atom parameters constrained
*S* = 1.01	*w* = 1/[σ^2^(*F*_o_^2^) + (0.0502*P*)^2^ + 0.0032*P*] where *P* = (*F*_o_^2^ + 2*F*_c_^2^)/3
1139 reflections	(Δ/σ)_max_ < 0.001
190 parameters	Δρ_max_ = 0.13 e Å^−^^3^
2 restraints	Δρ_min_ = −0.13 e Å^−^^3^

### Fractional atomic coordinates and isotropic or equivalent isotropic displacement parameters (Å^2^)

**Table:**

	*x*	*y*	*z*	*U*_iso_*/*U*_eq_	
O1	0.4119 (4)	0.1516 (3)	0.04946 (8)	0.0445 (5)	
O2	0.7298 (5)	0.4086 (3)	0.01338 (9)	0.0511 (6)	
O3	0.7306 (7)	0.7164 (3)	−0.03192 (13)	0.0742 (8)	
O4	0.6010 (5)	0.2116 (3)	0.14674 (10)	0.0581 (6)	
O5	0.4721 (8)	0.2569 (4)	0.24282 (12)	0.0859 (9)	
C1	0.6000 (7)	0.2076 (5)	0.00288 (13)	0.0401 (7)	
H1	0.7583	0.1044	−0.0022	0.048*	
C2	0.6456 (7)	0.5423 (5)	−0.03175 (14)	0.0494 (8)	
C3	0.4189 (7)	0.2325 (4)	−0.05400 (13)	0.0414 (7)	
C4	0.4482 (7)	0.4308 (5)	−0.07433 (13)	0.0445 (7)	
C5	0.3011 (8)	0.4956 (6)	−0.12687 (14)	0.0555 (9)	
H5	0.3231	0.6295	−0.1409	0.067*	
C6	0.1225 (9)	0.3563 (6)	−0.15739 (15)	0.0620 (10)	
H6	0.0207	0.3965	−0.1925	0.074*	
C7	0.0922 (9)	0.1584 (6)	−0.13675 (16)	0.0598 (9)	
H7	−0.0305	0.0669	−0.1582	0.072*	
C8	0.2407 (7)	0.0915 (5)	−0.08456 (14)	0.0499 (8)	
H8	0.2207	−0.0430	−0.0708	0.060*	
C9	0.5534 (7)	0.0616 (5)	0.10002 (14)	0.0443 (7)	
H9	0.7448	−0.0014	0.0890	0.053*	
C10	0.4522 (8)	0.1563 (5)	0.19777 (15)	0.0561 (9)	
C11	0.3520 (6)	−0.0951 (4)	0.12751 (13)	0.0383 (7)	
C12	0.2908 (6)	−0.0361 (5)	0.18517 (12)	0.0398 (7)	
C13	0.1088 (8)	−0.1539 (6)	0.22104 (14)	0.0527 (8)	
H13	0.0671	−0.1132	0.2601	0.063*	
C14	−0.0088 (8)	−0.3326 (5)	0.19756 (16)	0.0567 (9)	
H14	−0.1309	−0.4152	0.2209	0.068*	
C15	0.0531 (8)	−0.3905 (5)	0.13930 (15)	0.0565 (9)	
H15	−0.0294	−0.5119	0.1240	0.068*	
C16	0.2336 (7)	−0.2733 (5)	0.10336 (14)	0.0475 (8)	
H16	0.2740	−0.3131	0.0642	0.057*	

### Atomic displacement parameters (Å^2^)

**Table:**

	*U*^11^	*U*^22^	*U*^33^	*U*^12^	*U*^13^	*U*^23^
O1	0.0407 (11)	0.0523 (13)	0.0405 (11)	0.0026 (9)	0.0024 (9)	0.0118 (10)
O2	0.0571 (13)	0.0440 (13)	0.0520 (12)	−0.0096 (11)	−0.0019 (10)	0.0043 (11)
O3	0.097 (2)	0.0396 (14)	0.0862 (17)	−0.0174 (15)	0.0044 (15)	0.0040 (13)
O4	0.0705 (15)	0.0480 (13)	0.0551 (14)	−0.0231 (12)	−0.0137 (11)	0.0084 (11)
O5	0.132 (3)	0.0649 (18)	0.0596 (15)	−0.0248 (17)	−0.0195 (16)	−0.0118 (14)
C1	0.0425 (17)	0.0349 (15)	0.0430 (16)	−0.0007 (13)	0.0061 (13)	0.0023 (13)
C2	0.055 (2)	0.0401 (19)	0.0534 (18)	−0.0032 (15)	0.0119 (16)	0.0017 (16)
C3	0.0455 (17)	0.0412 (17)	0.0382 (15)	0.0043 (15)	0.0114 (13)	−0.0021 (14)
C4	0.0517 (18)	0.0407 (17)	0.0416 (16)	0.0028 (15)	0.0144 (14)	0.0031 (14)
C5	0.070 (2)	0.0505 (19)	0.0468 (18)	0.0112 (18)	0.0135 (17)	0.0141 (17)
C6	0.068 (2)	0.076 (3)	0.0421 (19)	0.012 (2)	0.0024 (17)	0.0031 (18)
C7	0.060 (2)	0.073 (3)	0.0467 (18)	−0.0015 (18)	0.0011 (16)	−0.0067 (19)
C8	0.056 (2)	0.0453 (19)	0.0483 (18)	−0.0011 (16)	0.0072 (15)	−0.0006 (15)
C9	0.0421 (16)	0.0425 (18)	0.0479 (17)	−0.0022 (14)	−0.0052 (13)	0.0075 (14)
C10	0.072 (2)	0.0447 (19)	0.051 (2)	−0.0063 (17)	−0.0157 (18)	0.0046 (17)
C11	0.0325 (14)	0.0384 (17)	0.0437 (16)	0.0012 (13)	−0.0046 (12)	0.0066 (13)
C12	0.0415 (15)	0.0410 (17)	0.0367 (15)	−0.0018 (14)	−0.0058 (12)	0.0053 (13)
C13	0.0552 (19)	0.064 (2)	0.0389 (16)	−0.0008 (18)	−0.0001 (14)	0.0066 (16)
C14	0.054 (2)	0.056 (2)	0.060 (2)	−0.0156 (17)	0.0015 (17)	0.0150 (17)
C15	0.062 (2)	0.0431 (18)	0.064 (2)	−0.0142 (17)	−0.0052 (17)	0.0016 (17)
C16	0.0485 (19)	0.0449 (18)	0.0490 (18)	−0.0032 (15)	−0.0011 (15)	−0.0036 (15)

### Geometric parameters (Å, °)

**Table:**

O1—C1	1.394 (3)	C6—H6	0.9300
O1—C9	1.403 (4)	C7—C8	1.391 (5)
O2—C2	1.372 (4)	C7—H7	0.9300
O2—C1	1.444 (4)	C8—H8	0.9300
O3—C2	1.192 (4)	C9—C11	1.495 (4)
O4—C10	1.374 (4)	C9—H9	0.9800
O4—C9	1.436 (4)	C10—C12	1.465 (5)
O5—C10	1.197 (4)	C11—C12	1.371 (4)
C1—C3	1.491 (4)	C11—C16	1.375 (4)
C1—H1	0.9800	C12—C13	1.380 (4)
C2—C4	1.467 (4)	C13—C14	1.371 (5)
C3—C4	1.372 (4)	C13—H13	0.9300
C3—C8	1.379 (4)	C14—C15	1.382 (5)
C4—C5	1.390 (4)	C14—H14	0.9300
C5—C6	1.373 (5)	C15—C16	1.375 (4)
C5—H5	0.9300	C15—H15	0.9300
C6—C7	1.372 (5)	C16—H16	0.9300
			
C1—O1—C9	116.0 (2)	C7—C8—H8	121.3
C2—O2—C1	110.6 (2)	O1—C9—O4	110.6 (2)
C10—O4—C9	110.7 (2)	O1—C9—C11	110.4 (2)
O1—C1—O2	111.1 (2)	O4—C9—C11	104.2 (2)
O1—C1—C3	109.6 (2)	O1—C9—H9	110.5
O2—C1—C3	104.2 (2)	O4—C9—H9	110.5
O1—C1—H1	110.6	C11—C9—H9	110.5
O2—C1—H1	110.6	O5—C10—O4	121.4 (3)
C3—C1—H1	110.6	O5—C10—C12	130.7 (3)
O3—C2—O2	121.4 (3)	O4—C10—C12	107.9 (3)
O3—C2—C4	130.6 (3)	C12—C11—C16	121.1 (3)
O2—C2—C4	108.0 (2)	C12—C11—C9	109.0 (3)
C4—C3—C8	121.3 (3)	C16—C11—C9	129.8 (3)
C4—C3—C1	109.1 (3)	C11—C12—C13	121.1 (3)
C8—C3—C1	129.6 (3)	C11—C12—C10	108.1 (3)
C3—C4—C5	120.9 (3)	C13—C12—C10	130.8 (3)
C3—C4—C2	108.1 (3)	C14—C13—C12	118.2 (3)
C5—C4—C2	130.9 (3)	C14—C13—H13	120.9
C6—C5—C4	118.1 (3)	C12—C13—H13	120.9
C6—C5—H5	121.0	C13—C14—C15	120.3 (3)
C4—C5—H5	121.0	C13—C14—H14	119.9
C7—C6—C5	120.8 (3)	C15—C14—H14	119.9
C7—C6—H6	119.6	C16—C15—C14	121.7 (3)
C5—C6—H6	119.6	C16—C15—H15	119.2
C6—C7—C8	121.5 (4)	C14—C15—H15	119.2
C6—C7—H7	119.2	C11—C16—C15	117.5 (3)
C8—C7—H7	119.2	C11—C16—H16	121.2
C3—C8—C7	117.3 (3)	C15—C16—H16	121.2
C3—C8—H8	121.3		

### Hydrogen-bond geometry (Å, °)

**Table:**

*D*—H···*A*	*D*—H	H···*A*	*D*···*A*	*D*—H···*A*
C6—H6···O5^i^	0.93	2.68	3.407 (4)	136
C1—H1···O3^ii^	0.98	2.61	3.337 (4)	131

Symmetry codes: (i) *x*−1/2, −*y*+1, *z*−1/2; (ii) *x*, *y*−1, *z*.
